# Low Intakes of Iodine and Selenium Amongst Vegan and Vegetarian Women Highlight a Potential Nutritional Vulnerability

**DOI:** 10.3389/fnut.2020.00072

**Published:** 2020-05-20

**Authors:** Naomi Fallon, Stephanie A. Dillon

**Affiliations:** School of Sport and Health Sciences, University of Central Lancashire, Preston, England

**Keywords:** vegan, vegetarian, iodine, selenium, female, micronutrients, diet

## Abstract

Vegan and vegetarian diets are becoming increasingly popular in the UK. Due to the avoidance of animal products there can be significant differences in nutrient intakes between meat-eaters and vegetarians, and especially vegans. Importantly, research has identified that both vegans and vegetarians may be vulnerable to low intakes of some micronutrients. The aim of this study was to investigate micronutrient intake in omnivorous, vegetarian and vegan women. In total, 62 women (26 omnivores, 16 vegetarians, 20 vegans, mean age 31.6 ± 12.4 y, mean BMI 24.1 ± 1.6 kg/m^**2**^) completed 4-day diet diaries. Diet intake data was analyzed using Nutritics and nutrient intake levels were compared with national dietary recommendations (RNIs). Statistical analysis was performed using SPSS, with differences between the groups identified using ANOVA with *post-hoc* Bonferroni correction. All groups recorded intakes of vitamin D, iron, iodine and selenium below RNI. The vegan group had significantly lower intakes of vitamin D, vitamin B12, calcium, selenium and iodine than vegetarians and omnivores (*p* < 0.05), with particularly low intakes of selenium (24.7 ± 11.9 μg) and iodine (24.4 ± 12.7 μg). These results suggest that adult women in the UK are at risk of low intakes of several vitamins and minerals, with the exclusion of animal products conferring an additional vulnerability, particularly with respect to selenium and iodine, both of which play important roles in thyroid hormone production. This study highlights iodine and selenium intakes to be a concern amongst women who follow vegan diets, and the necessity of further research to identify if low intake translates to biochemical markers and functional status.

## Introduction

In recent years the UK has witnessed an increase in the availability and popularity of animal-free food and drink. In 2018, one in six food products launched in the UK claimed to be vegan or contain no animal ingredients (1). Sales of non-dairy milks increased by 9.4% between 2016 and 2017 (1) whilst UK supermarket chain Sainsburys report a 65% increase in sales of plant-based products year-on-year (2). These trends are driven by consumers, with as many as one third (33.5%) reporting that they are cutting down on or cutting out meat altogether, with the majority (21%) adopting a flexible or “flexitarian approach,” 9.5% becoming vegetarian and 3% vegan (3). Nationally, the number of people adopting vegetarian and vegan diets in the UK are estimated to be 3 and 1%, respectively (4), and higher in university students with 3% self-identified as vegan and 8% as vegetarian (5). Health is the primary motivation cited for reducing meat intake, both in reaction to health scares and efforts to improve overall health, with concerns over meat production, including animal cruelty and the climate change impact also commonly cited (5, 6).

Vegetarian diets are generally classed as those that include eggs and dairy but exclude meat, poultry and fish, whereas individuals following a vegan diet do not consume any food of animal origin (7). Due to the avoidance of animal products, there can be striking differences in nutrient intakes between meat-eaters and vegetarians, and especially vegans (8, 9). Vegetarians and vegans may have a lower BMI (10) and higher overall diet quality (7, 11) than meat eaters, with changing to a vegan diet resulting in a lower protein and fat intake, including saturated fat (12) and higher intake of dietary fiber (8–10). Micronutrient intake can also differ significantly, as vegans may have higher intakes of vitamins C, E, B1, folate, magnesium and iron, but lower intakes of retinol, vitamin D, vitamin B12, calcium and zinc (8). Iron intake has been found to be both lower (13) and higher in vegans than meat eaters (8, 9), and a higher intake has not been found to translate to a higher plasma ferritin level (14), suggesting a complex picture.

Cross-sectional studies of self-reported dietary intake have previously focused solely on the implications of nutrient intake as it relates to dietary lifestyle and involved both male and female participants. However, when considering the nutritional adequacy of a diet, it is important to consider that the micronutrient intakes of adult males and females in the UK can also differ significantly. A secondary analysis of the latest data from the National Diet and Nutrition Survey (NDNS) revealed that UK females had significantly lower intakes of 9 micronutrients (riboflavin, vitamin B6, B12, folic acid, calcium, iron, magnesium, potassium, and iodine) than their male counterparts (*p* < 0.001) (15).

The nutritional status for women of child-bearing age has ramifications that can span generations (16) which compound the concerns over the nutritional adequacy of the diets of female adolescents and women of child-bearing age, particularly in respect to the minerals iron, selenium and iodine. Adult women fail to meet the Reference Nutrient Intake (RNI) for iron, with 23% recording intakes below the Lower Reference Nutrient Intake (LRNI) levels and 15.5% having low plasma ferritin levels. Over half (51%) of adult women had intake of selenium below the LRNI of 40 μg/day whilst the mean urinary iodine concentration was 117 μg/L (17), lower than the 150 μg/L recommendation. Iodine is predominantly found in fish and milk, is rarely fortified in plant-alternatives to milk (18), and the lack of vegan or vegetarian dietary sources of iodine suggest a heightened vulnerability of women following diets free from animal-based foods. However, whether following a particular diet exasperates the risk of nutritional deficiencies amongst women is not clear.

In this article we report the results from an analysis of nutrient intakes in adult women following omnivorous, vegan or vegetarian diets. The main aim of this study was to identify whether adult women in England following omnivorous, vegan or vegetarian diets were vulnerable to insufficient intakes in key micronutrients.

## Methods

### Recruitment

Participants were initially recruited on campus and through the dissemination of posters, and off campus by word of mouth and on personal social media networks. Further recruitment was achieved via the university student vegan society and social media channels where local vegan and vegetarian groups were targeted to achieve an even sample spread. All participants provided written informed consent and ethical approval to conduct this study was granted by the University of Central Lancashire.

### Lifestyle Characteristics and Anthropometry

The four-day diet diary included a section where participants were asked to report their age, height and weight and whether they were vegan or vegetarian or omnivores. The diaries were cross-checked to ensure they had correctly identified themselves.

### Food Diary and Diet Analysis

Participants were asked to complete a diary of everything they ate and drank over a four-day period, which included one weekend day. The front of the diet diaries included detailed direction on how to complete the diaries, including pictorial estimates of portion sizes and guidance on weighing food. Participants were encouraged to be as detailed as possible, including information on the brand of product used and cooking methods. This study was concerned only with nutrient intake from food sources, therefore participants were not asked about their supplement use, and any information on supplements provided by participants was not included in the analysis. Participants were invited to contact the researcher if they had any questions or queries relating to completion of the food diary.

The completed diet diaries were input into Nutritics nutrition analysis software (version 4.315 Education). Where manufactured food items were not already available within the software, the researcher sought out the official packaging online, from which the nutritional information was manually input. For meals created by the participant from a variety of base ingredients, the meal was recreated in the software.

### Data Analysis

A comprehensive analysis of the nutrient intake data was undertaken using Statistical Package of Social Science (IBM SPSS version 24, Chicago, IL, USA). All data is presented alongside the Standard Deviation (±SD).

Mean nutrient intakes for the groups were compared with the current UK Dietary Reference Values (DRV) for adult females in the form of Reference Nutrient Intakes (RNIs) (19). Estimated Average Requirements (EAR) are used for energy and some nutrients in the absence of an RNI.

One-way ANOVA with *post hoc* Bonferroni correction was employed to assess differences in dietary intake between the three groups. The level of significance was set at *p* < 0.05. In the absence of information on physical activity levels, the current Reference Nutrient Intakes (RNIs) for moderately active adult females were used for comparison (20).

## Results

### Subject Characteristics

In total, 62 female participants completed the study. The youngest participant was 18 years and the oldest was 67 years, with a mean age of 32.6 ± 13.7 years. (Table 1) gives an overview of the participant characteristics (±SD) by group.

**Table 1:** Participant characteristics by group.

	**All** **(*****n*** **=** **62)**		**Omnivore** **(*****n*** **=** **26)**		**Vegetarian** **(*****n*** **=** **16)**		**Vegan** **(*****n*** **=** **20)**		
	**Mean**	**SD**	**Mean**	**SD**	**Mean**	**SD**	**Mean**	**SD**	***P*-value**
Age (years)	31.6	12.4	32.2	12.6	35.9	15.9	27.6	7.7	0.138
BMI (kg/m^2^)	24.1	1.6	24.6	2.2	24.4	1.4	23.8	1.1	0.509

Of the 62 female participants, 26 were omnivores (mean age *32.2* ± *12.6* years), 16 were vegetarian (mean age *35.9* ± *15.9* years), and there were 20 vegans (mean age *27.6* ± *7.7* years).

The three groups did not significantly differ in age. All groups had a self-reported height and weight which put them in the healthy BMI range of 18.5–24.9 kg/m^2^ (21).

### Energy and Macronutrient Intake

Table 2 presents the mean (±SD) intake of energy and macronutrients across the different diets along with the results of the between groups ANOVA. Total calorie intake was below the estimated average requirement for all groups, whilst all groups met the RNI for protein and were within recommended levels for total fat (78 g/day), and saturated fat (24 g/day). The omnivore group (31.6 ± 19.8 g/day) exceeded the RNI for free sugars of 27 g/day. With an average intake of 35.3 ± 7.1 g/day, the vegan group was the only group to meet the recommended 30 g/day for dietary fiber.

**Table 2:** Daily energy, macronutrient, and fiber intakes across the three diet groups.

**Nutrient**	**DRV**	**All** **(*****n*** **=** **62)**		**Omnivore** **(*****n*** **=** **26)**		**Vegetarian** **(*****n*** **=** **16)**		**Vegan** **(*****n*** **=** **20)**		
		**Mean**	**SD**	**Mean**	**SD**	**Mean**	**SD**	**Mean**	**SD**	***P*-value**
Energy (Kcal)	2,000^*^	1,778.2	379.0	1,857.8	389.7	1,704.1	401.9	1,734.2	344.1	0.369
Protein (g)	45	73.3	25.0	90.1^a, b^	27.3	60.8^a^	14.3	61.6^b^	14.1	<0.001
Total fat (g)	78	68.0	22.1	72.3	26.3	64.9	14.7	64.9	21.1	0.431
Sat fat (g)	24	20.6	9.8	23.9^a^	10.3	23.2^b^	9.1	14.2^a, b^	6.2	0.001
Carbohydrates (g)	267	211.1	59.4	206.7	58.4	215.4	70.9	213.3	53.2	0.886
Free Sugars (g)	27	27.0	24.1	31.6	19.8	22.5	14.5	24.7	24.3	0.432
Fiber (g)	30	28.3	10.5	22.7^a^	7.5	28.4	13.3	35.3^a^	7.1	<0.001

* *Estimated average requirement for adult females (22)*.

*P-value represents result of between groups ANOVA*.

a, b *Means with a superscript represent significant difference (p < 0.05) identified in post-hoc analysis*.

There was a significant difference in protein intake between the omnivore group and both the vegetarian and vegan groups. The intake of saturated fat was significantly higher in the omnivore and vegetarian groups than in the vegan group, and dietary fiber intake was significantly lower in the omnivore group than in the vegan group.

### Micronutrient Intake

(Table 3) presents the mean (±SD) intake of micronutrients across the different dietary groups and result of the between groups ANOVA. All three groups reported sufficient intakes of vitamins A, C, E, and the B vitamins thiamine, riboflavin, niacin, folates and vitamin B6. The three groups also met the RNI for zinc and were within the recommended intake level for sodium of 2,400 mg.

**Table 3:** Daily micronutrient intakes for the three diet groups.

**Nutrient**	**DRV**	**All (*****n*** **=** **62)**		**Omnivore (*****n*** **=** **26)**		**Vegetarian (*****n*** **=** **16)**		**Vegan (*****n*** **=** **20)**		***P*-value**
		**Mean**	**SD**	**Mean**	**SD**	**Mean**	**SD**	**Mean**	**SD**	
Vit A (μg)	600	978.0	936.9	1,004.3	932.8	805.8	493.5	1,081.7	1,201.7	0.675
Vit C (mg)	40	119.6	144.2	118.2	204.0	105.7	79.0	132.5	81.9	0.859
Vit D (μg)	10	2.6	1.7	3.5^a^	1.7	2.3	1.2	1.6^a^	1.2	<0.001
Vit E (mg)	4^*^	10.1	4.3	9.7	3.7	9.3	5.2	11.2	4.3	0.355
Vit K (μg)	90	85.6	136.3	60.7	104.9	57.3	63.8	140.7	192.9	0.088
Vit B12 (μg)	1.5	3.1	2.3	4.8^a, b^	2.2	2.5^a^	1.6	1.3^b^	0.8	<0.001
Thiamine (mg)	0.8	1.8	1.0	1.6	0.6	1.6	0.6	2.1	1.4	0.090
Riboflavin (mg)	1.1	1.4	0.6	1.7^a^	0.7	1.3	0.6	1.2^a^	0.5	0.027
Niacin (mg)	13.2	30.1	13.4	40.0^a^	12.6	23.4^a^	8.7	22.7	9.0	<0.001
Pantothenic Acid (mg)	5^*^	5.7	5.3	5.8	2.0	4.1	1.7	6.9	8.9	0.292
Vit B6 (mg)	1.2	1.7	0.8	2.0	1.0	1.4	0.6	1.6	0.4	0.049
Folate (μg)	200	288.6	140.4	253.5^a^	115.0	262.3	124.3	355.2^a^	163.6	0.033
Biotin (ug)	30^*^	33.7	16.3	34.7	15.2	34.5	19.2	31.8	15.9	0.302
Sodium (mg)	2,400	2,003.7	641.8	2,052.3	741.7	2,104.6	550.8	1,859.9	570.5	0.468
Potassium (mg)	3,500	2,812.7	812.8	2,839.1	649.2	2,775.6	1,128.7	2,808.0	747.5	0.971
Calcium (mg)	700	727.3	279.3	806.6^a^	314.1	786.3	265.0	577.1^a^	173.1	0.011
Iron (mg)	14.8	12.2	4.4	11.9	4.1	10.7	5.0	13.9	4.1	0.088
Iodine (μg)	150	79.4	62.5	112.6^a^	62.1	90.8^b^	55.9	24.4^a, b^	12.7	<0.001
Selenium (μg)	60	41.9	22.9	57.0^a, b^	21.7	38.7^a^	19.0	24.7^b^	11.9	<0.001
Zinc (mg)	7	8.4	3.5	8.9	2.3	7.7	2.7	8.3	5.2	0.536

* *AI set in absence of RNI*.

*P-value represents result of between groups ANOVA*.

a, b *Means with a superscript represent significant difference (p < 0.05) identified in post-hoc analysis*.

All three groups recorded intakes below recommended levels (RNI) for potassium, vitamin D, iron, selenium and iodine. For iodine, the vegan group's intake of 24.4 μg/day was below the lower reference nutrient intake (LRNI) level of 70 μg/day (22), and both the vegetarian group and vegan group failed to reach the LRNI for selenium of 40 μg/day, at 38.7 ± 19 and 24.7 ± 11.9 μg/day, respectively. Additionally, the vegan group failed to reach RNI for vitamin B12 or calcium, whilst both the vegetarian groups and omnivore group failed to reach the RNI for vitamin K.

*Post-hoc* analysis revealed significant differences in micronutrient intakes between the groups. Compared with the vegetarian group, the omnivore group reported significantly higher intakes of vitamin B12, niacin and selenium. Compared with the vegan group, the omnivore group reported significantly higher intakes of vitamin B12, niacin, riboflavin, vitamin D, calcium and selenium, and significantly lower intake of folates. The intake of iodine reported by the vegan group was significantly lower than both the omnivore and vegetarian groups.

## Discussion

This study identified that there is substantial variability in the dietary intake of adult women in the UK following different dietary lifestyles, and that adhering to a vegan diet may exacerbate their vulnerability to inadequate intakes of key vitamins and minerals, most notably vitamin B12, calcium, selenium and iodine.

The findings from this study on the macronutrient intakes of omnivores, vegetarians and vegans are consistent with previous studies which show a tendency for vegans to have a diet that is lower in protein, total fat and saturated fat intake than omnivores (12, 17) and higher in dietary fiber (9, 10).

Despite the considerable differences between the groups, the macronutrient intakes of all three groups were largely consistent with current government recommendations for a healthy diet (19), with the exception of dietary fiber, of which only the vegan groups achieved the recommended 30 g/day. A higher consumption of dietary fiber is associated with lower incidence of coronary heart disease, stroke, type 2 diabetes and colorectal cancer, and decrease in cardiovascular and stroke related mortality, and all-cause mortality (23). The effects of dietary components on health are complex however, and components of dietary fiber such as phytates are thought to bind to certain minerals, reducing their absorption (24), although the regular consumption of a high phytate diet could potentially lessen their inhibitory effect (25). Two minerals considered to be vulnerable to losses are calcium, and iron, both of which the vegan group averaged below guideline intake.

Indeed, all three groups recorded an average iron intake that was below the RNI of 14.8 mg, which is especially important for women of childbearing age due to menstrual losses (16). The vegan group had a higher average intake, but this does not necessarily translate to a higher plasma ferritin level, which may be the result of the predominance of non-heme iron in plant sources (14). Non-heme iron has a lower bioavailability than heme iron found in animal sources, perhaps as little as 2–20%, although absorption is enhanced with the concurrent consumption of ascorbic acid (24, 26), for which all three groups had sufficient intakes. A systematic review by Haider et al. (27) found that the difference in iron status between women consuming vegetarian or omnivorous diets was unclear, with half of studies reporting no difference (11 of 22 studies) and significance decreasing when studies with a high risk of bias were excluded.

In line with previous studies [e.g., (8, 28)], the vegan group had significantly lower intakes of vitamin B12 than both the vegetarian and omnivore groups, and it remained below the RNI even with the consumption of fortified plant milks. There was a low intake of vitamin D across the groups, with the intake amongst the vegan group significantly lower than that of the omnivore group. When combined with the low intake of calcium amongst vegans, there is the potential negative implications for bone health. Indeed, there is evidence to suggest that vegans may have higher levels of circulating markers for bone turnover (29).

Arguably the most striking findings to emerge from this explorative study were the extremely low reported intakes of iodine and selenium for vegans, both of which were below their respective LRNIs, at <11 and 50% of daily recommended amounts. For the omnivore group, the predominant sources of both iodine and selenium were meat, eggs, fish (such as salmon, tuna, haddock) and dairy, and for the vegetarian group it was dairy products, such as yogurt, cheese and milk. This suggests that diets which exclude meat and dairy products are particularly vulnerable to low intakes and may rely on fortified products or supplementation. Indeed, a recent study conducted by Schüpbach et al. (14) reported a median urinary concentration of iodine of 56 μg/l in vegans, far below WHO guideline of 100 μg/l and which occurred despite salt iodisation.

In this present study 78% of the adult women were of childbearing age (18–45 y). This proportion was highest for the vegan group (95%) and lowest for the vegetarian group (69%). Both selenium and iodine are essential for healthy thyroid function and of particular importance for pregnant women or women who may become pregnant, due to their role in healthy fetal development. Indeed, mild iodine deficiency has been found in the UK both in women of child-bearing age (30) and during pregnancy (31), and in pregnant women elsewhere in Europe, the use of multivitamins (32) or iodised salt (33) have not been found to reliably increase iodine concentrations to adequate levels.

The methods of assessment chosen as 4 day weighed diet diaries is a common and accepted form of assessment of intake and sampling was opportunistic with participants responding of their own volition. The inclusion of weighed records aid accuracy or the recording and they do not rely on memory. However, self-perception of intake is still often erroneous, with widespread underreporting even over a 7-day period (34), and the recording process may lead to a change in their usual eating pattern. The inclusion of a second method of analysis; combining food records with biomarker analysis would increase the reliability of the results.

In addition, this study was assessing only the availability of nutrients through dietary sources and consequently participants were not asked to provide information on supplement use. The results suggest a low availability of dietary sources of vitamin B12, vitamin D, calcium, iodine and selenium in the vegan diet, and consequently highlight the important role for supplementation. There are a large range of single and multi-nutrient supplements available, all of which vary considerably in terms of dose and consistent use (28). Future research into the sufficiency of the current supplemental regime of vegans and vegetarians would be beneficial to gain a more thorough understanding of the current situation.

## Conclusion

Adhering to a vegan diet was associated with lower intakes of key micronutrients including vitamin B12, vitamin D, calcium, iodine and selenium in a sample of adult women in the UK. The extremely low reported intakes of iodine and selenium amongst the vegetarians and especially vegans, combined with their limited food availability in diets which omit animal foods, highlights them both as areas of concern. Furthermore, the wide disparities in intakes between the dietary groups suggest that cross-sectional and cohort studies that do not distinguish between dietary lifestyles may not be providing an accurate picture.

## Data Availability Statement

The datasets generated for this study are available on request to the corresponding author.

## Ethics Statement

The studies involving human participants were reviewed and approved by The University of Central Lancashire. The patients/participants provided their written informed consent to participate in this study.

## Author Contributions

NF conceived and designed the study, analyzed the data, and was lead writer of the paper. SD contributed to conception and design of the study, interpretation of data, and provided critical feedback on the manuscript. All authors contributed to manuscript revision, read, and approved the submitted version.

## Conflict of Interest

The authors declare that the research was conducted in the absence of any commercial or financial relationships that could be construed as a potential conflict of interest.
